# Neuroimmune Activation in a Goat Model of Intervertebral Disc Degeneration

**DOI:** 10.3390/cells15030286

**Published:** 2026-02-03

**Authors:** Janai A. Augustin, Kevin G. Burt, Caitlin Barrett, Matthew Fainor, Brianna S. Orozco, Thomas P. Schaer, Harvey E. Smith, Robert L. Mauck, Sarah E. Gullbrand

**Affiliations:** 1 Department of Orthopaedic Surgery, Perelman School of Medicine, University of Pennsylvania, Philadelphia, PA 19104, USA; 2 Translational Musculoskeletal Research Center, CMC Veterans Affairs Medical Center, Philadelphia, PA 19104, USA; 3 Department of Bioengineering, University of Pennsylvania, Philadelphia, PA 19104, USA; 4 CReATE Motion Center, CMC Veterans Affairs Medical Center, Philadelphia, PA 19104, USA; 5 Drexel University College of Medicine, Philadelphia, PA 19129, USA; 6 Department of Clinical Studies, New Bolton Center, School of Veterinary Medicine, University of Pennsylvania, Philadelphia, PA 19104, USA

**Keywords:** neuroinflammation, pre-clinical models, intervertebral disc degeneration, inflammation, goat cervical spine, adjacent segment degeneration, peripheral sensitization, dorsal root ganglion

## Abstract

Intervertebral disc degeneration (IVDD) initiates a cascade of structural and biological changes that compromise mechanical function, often leading to chronic pain. While small animal models have provided insight into inflammatory and nociceptive mechanisms of IVDD, translational studies require large animal models that more closely replicate human spine anatomy and physiology. This study induced cervical disc degeneration via intradiscal chondroitinase ABC (ChABC) injection in a large animal model and evaluated the associated disc pathology and neuroinflammatory responses across IVDs and within spinal cord and dorsal root ganglia (DRG) tissues. Results confirmed structural degeneration at ChABC-injected levels and revealed additional evidence of adjacent segment degeneration. Neuroinflammatory analyses revealed innervation, via deposition of PGP9.5 and NFH, throughout both ChABC-injected and adjacent IVDs. Monocyte markers were significantly increased in ChABC-degenerated IVDs. Across experimental groups, the level of monocyte (Ly6C) and macrophage (CD68) markers correlated with worsened histological scores and with reduced mechanical integrity. Similarly, increased production of the neuropeptide, Substance P, in IVDs was significantly positively correlated with compromised IVD mechanical function. Finally, we observed elevated production of the microglia marker, Iba1, and Substance P production in the spinal cord, with similar trends in DRGs, in degenerative spines. By establishing quantitative relationships between disc pathology, immune responses, and neural activation, this work established possible disease-contributing neuroinflammatory activation and further validated a clinically relevant model for preclinical evaluation of regenerative and therapeutic strategies.

## 1. Introduction

Intervertebral disc degeneration (IVDD) is a multifactorial condition that underlies a significant proportion of chronic back pain and disability, making it a leading cause of musculoskeletal morbidity worldwide [1]. IVDD is characterized by a cascade of structural and biological changes that compromise disc mechanical function and height, often culminating in discogenic pain [2]. In a healthy disc, a highly hydrated gel-like nucleus pulposus (NP) is bounded by an outer annulus fibrosus (AF) formed by concentric lamellae of collagen fibers, along with superior and inferior cartilaginous endplates that serve as interfaces with adjacent vertebral bodies [3]. The intricate interplay between matrix and cellular turnover and mechanical loading within the disc renders its degeneration a difficult process to study in vitro or using small animal models. These models often fail to recapitulate the human condition due to marked differences in disc size, geometry, and mechanical environment [4]. Given these limitations, large animal models have emerged as an intermediate translational platform that better mimics human disc pathophysiology and enables evaluation of disease progression, imaging biomarkers, biomechanics, and therapeutic responses.

The goat cervical spine is an ideal model for studying intervertebral disc degeneration because disc geometry and biomechanical loading closely approximate those of humans, despite the quadrupedal posture of the animal [5]. In addition, adult goats lack notochordal cells, mirroring the human condition in which early loss of these cells predisposes the disc to degeneration, and disc proteoglycan and collagen composition provides an extracellular matrix environment similar to that of humans [5]. Intradiscal injection of chondroitinase ABC (ChABC) reliably induces degenerative changes in the goat that recapitulate hallmark features of human disc disease, making it a robust and reproducible model for translational studies [2,5]. Our previous work showed that intradiscal injection of ChABC into the goat cervical intervertebral disc produced reproducible degrees of degeneration that extended to adjacent endplates and facet joints, thereby recapitulating whole motion segment pathology [5]. Enzymatic degradation of proteoglycans in the nucleus pulposus led to reduced hydration, loss of disc height, diminished T2 values on MRI, annulus fibrosus disorganization, and decreased cellularity 12 weeks post ChABC delivery. These disc changes were accompanied by vertebral endplate remodeling, characterized by increased bone density and focal resorptions resembling Schmorl’s nodes, which altered small-molecule transport across the endplate [5]. Adjacent facet joints also exhibited early osteoarthritic changes, with reductions in cartilage mechanical properties occurring prior to pronounced histological degeneration. The severity of disc degeneration correlated with alterations in endplate diffusion and facet cartilage biomechanics, highlighting crosstalk across spinal tissues [5]. While this prior study illustrated the complex structural changes that occur in the disc and associated structures in this model, it did not investigate the neuroinflammatory mechanisms involved in this process.

The intervertebral disc (IVD) is normally immune privileged and minimally innervated; however, degeneration is marked by persistent inflammation and aberrant nerve ingrowth that contributes to structural breakdown and discogenic pain. Pro-inflammatory cytokines (IL-1β, TNF, and IL-6) [6,7] orchestrate a catabolic shift in disc cells, promoting extracellular matrix degradation through the upregulation of matrix metalloproteinases and aggrecanases. Simultaneously, these cytokines stimulate the expression of neurotrophic factors that facilitate nociceptive ingrowth into previously aneural regions of the disc [8,9]. These changes compromise biomechanical integrity, disrupt nutrient transport, and establish a self-sustaining cycle of inflammation and innervation that underlies chronic low back pain. Rodent models have elucidated key inflammatory pathways, but a major gap remains in defining these processes in larger, translationally relevant systems, limiting our ability to evaluate therapeutic strategies targeting these inflammatory and neurogenic mechanisms [10,11,12].

Further complicating the identification of inflammatory changes within a large animal model is the need for a baseline comparator group of non-operated animals. These controls are paramount, as clinical observations have revealed strong associations between the severity of IVD disease at one level and the extent of disease regionally throughout spinal joints, including adjacent IVDs and facet joints [13]. Furthermore, recent evidence from small animal models of IVDD showed that discs adjacent to injury undergo significant inflammatory changes [14,15]. To most effectively utilize translational large animal models for testing inflammatory-focused therapies for treating spinal diseases and the associated pain, we must first identify the neuroinflammatory mechanisms that underlie spinal joint diseases.

Addressing these gaps requires the characterization of structural degeneration, inflammation, and the development of reliable methods for assessing pain, since pain relief remains the primary clinical success criteria in patients. Pain can be directly assessed in small animal models and reliably correlates with IVDD severity [16,17]. However, no validated quantitative metrics of musculoskeletal pain currently exist for large animal models, particularly goats, presenting a challenge for translational studies. Neurobiological markers in the dorsal root ganglia (DRG) and spinal cord provide quantifiable measures of neuroinflammation and nociceptive sensitization. Glial fibrillary acidic protein (GFAP) is a marker of astrocytic activation, and ionized calcium-binding adapter molecule 1 (Iba1) reflects microglial activation, both of which signify neuroinflammatory changes within the DRG and spinal cord [18,19]. Substance P is a neuropeptide associated with nociceptive neurons and represents a key mediator of pain signaling [20,21]. Together, these markers capture processes of neuroinflammation and neuronal sensitization, making them valuable surrogate measures of pain in preclinical models. Building on their established correlation with pain in a rat discogenic pain model [12], we sought to determine whether these markers are present in a large animal model of disc degeneration to establish mechanistic connections between disc degeneration and pain-related outcomes. We hypothesize that structural and functional changes observed in our large animal model of IVDD will correlate with increased innervation and immune cell infiltration into the disc, contributing to neuroinflammatory responses.

## 2. Materials and Methods

### 2.1. Induction of Disc Degeneration

**Figure 1:**
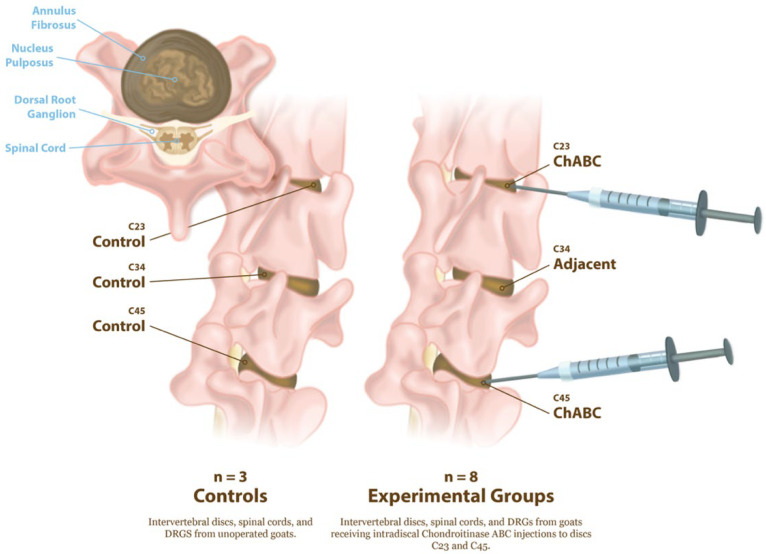
Experimental design for induction of intervertebral disc degeneration and tissue collection. Schematic illustration of cervical spine levels examined in control and ChABC-treated goats. Controls (n = 3) consisted of non-operative animals from which intervertebral discs, spinal cords, and DRGs at C2–C3, C3–C4, and C4–C5 were collected. Experimental animals (n = 8) received intradiscal Chondroitinase ABC (ChABC) injections at C2–C3 and C4–C5, generating degenerated discs and associated adjacent-level discs (C3–C4). Following 12 weeks of ChABC injection, IVDs, spinal cords, and DRGs were harvested for structural, mechanical, and neuroinflammatory analyses.

All animal procedures were approved by the Institutional Animal Care and Use Committee of the University of Pennsylvania (Protocol #803331, initial approval 10/2017, renewal approved 09/2025) and performed in accordance with established protocols for goat cervical spine surgery. Degeneration of the cervical intervertebral discs was induced in eight large frame goats (castrated males), approximately 2–5 years of age (73.7 ± 13.3 kg, Thomas D. Morris, Inc., Reisterstown, MD, USA), via ChABC injection. The degenerative changes to the intervertebral discs, facet joints, and vertebral endplates in these animals has been reported in our prior publication [5]. Animals were group-housed with unrestricted exercise in a barn with natural bedding for the duration of the study. Animals were sedated with diazepam (0.5–1.5 mg/kg, intravenous [IV]/intramuscular [IM]) or midazolam (0.3–0.5 mg/kg IV/IM), followed by induction of general anesthesia with ketamine (2.2–4.0 mg/kg, IV). Anesthesia was maintained with 1–5% isoflurane in oxygen. Animals were positioned in dorsal recumbency, and fluoroscopy was used to identify the C2–C3, C3–C4, and C4–C5 disc spaces. A 6-inch 22G spinal needle was inserted into the nucleus pulposus of the C2–C3 and C4–C5 discs via a percutaneous ventral approach under fluoroscopic guidance (Arcadis Orbis, Siemens, Munich, Germany) (Figure 1).

A total of either 2U or 5U Chondroitinase ABC (ChABC, Amsbio, Cambridge, MA, USA) suspended in 200 μL of sterile phosphate-buffered saline (PBS) containing 0.1% bovine serum albumin was injected into each disc. Each animal received one 2U and one 5U ChABC injection, with the levels randomized between the C2–C3 and C4–C5. The C3–C4 level served as an internal, adjacent control. Study animals received buprenorphine (0.005–0.01 mg/kg IV or IM) perioperatively (SID-QID) and/or fentanyl (2.5 mcg/kg/hr transdermal, removed after 72 h) and flunixin meglumine (1.1 mg/kg IV or IM, SID for 3 days beginning the day of surgery). All animals were examined twice daily by a veterinarian for neurologic deficits, gait, and general clinical well-being for the duration of the study. The humane endpoint criteria included prolonged inappetence in conjunction with severe weight loss, anorexia unresponsive to treatment, systemic signs of postoperative infection unresponsive to treatment, dyspnea/tachypnea unresponsive to treatment, severe depression/lethargy unresponsive to treatment, and severe musculoskeletal/neurological abnormalities. Of the eight animals initially enrolled, seven completed the full study protocol, while one was allocated to a separate pilot study and excluded from post-mortem analyses. All animals reached the study endpoint without clinical complications. Animals were euthanized via overdose of pentobarbital (Euthasol, 1 mL/10 lbs, I.V., to effect). Following euthanasia, the cervical spines were harvested, and the C2–C3, C3–C4, and C4–C5 motion segments were isolated. Three additional cervical spines from large frame goats were obtained from additional animals that had never undergone cervical spine surgery, to serve as healthy controls. Each motion segment was divided into the anterior column, consisting of the vertebral body–intervertebral disc–vertebral body unit and the posterior elements containing the facet joints. The spinal cord and DRG, located posterior to the vertebral column, were also collected (Figure 1).

### 2.2. In Vivo Magnetic Resonance Imaging (MRI) and Intervertebral Disc Biomechanical Testing

At 12 weeks post-operatively, animals underwent cervical spine MRI under general anesthesia. A T2-weighted CPMG sequence (TR = 3000 ms, TE = 13.6–340 ms, in-plane resolution = 0.56 mm × 0.56 mm, FoV = 325 mm, slice thickness = 5.0 mm) was used to quantify T2 relaxation times in the nucleus pulposus by fitting signal intensities to noise-corrected exponentials [22].

Following MRI, animals were euthanized and cervical spine motion segments were dissected into bone–disc–bone units, with facets removed. These motion segments underwent compressive mechanical testing on an Instron 5948 following our established protocols [2,23,24]. The cranial and caudal vertebral bodies were potted in a low-melting temperature alloy, and specimens were subjected to 20 cycles of compressive loading from −0.5 N to −100 N, followed by a 1 h creep hold at −100 N (~0.24 MPa). This loading magnitude is within the physiological range for both goat and human cervical spines [25]. All testing was performed in a PBS bath at room temperature. Axial displacement was measured optically by placing ink markers on each vertebral body adjacent to the disc and tracking their positions with a digital camera. Force and displacement data were normalized to stress and strain based on disc geometry obtained from MRI. Disc area was determined by segmenting pixels within the disc on sagittal and axial images and multiplying by in-plane resolution. Disc height was calculated from the midsagittal disc area divided by the anterior–posterior disc width. Stress was defined as applied force normalized to disc area, and strain as axial displacement normalized to original disc height. A bilinear fit of the 20th loading cycle was performed using MATLAB (R2025b) to identify toe and linear regions, yielding toe modulus, linear modulus, transition strain (intersection of fit lines), and maximal strain.

### 2.3. IVD Histopathology

Following mechanical testing, motion segments were fixed in 10% neutral buffered formalin at 4 °C for one week. The anterior motion segments were then decalcified (Formical 2000; Decal Chemical Corporation, Tallman, NY, USA), embedded in paraffin, and sectioned (10 µm) in the mid-sagittal plane. Sections were stained with Hematoxylin and Eosin to assess cell morphology and density. All stained slides were imaged at 20× magnification using an Aperio slide scanner (Leica Biosystems, Buffalo Grove, Lincolnshire, IL, USA). Histological evaluation was performed using the JOR Spine/ORS Spine Section grading system for large animal models [1].

### 2.4. Immunohistochemical Analysis for Neuro-Immune Inflammatory Response in Discs

Additional paraffin sections were deparaffinized and rehydrated with a series of ethanol washes. Antigen retrieval was performed using proteinase K (S302030-2, Agilent Technologies Inc., Santa Clara, CA, USA) at 37 °C for 4 min, followed by blocking with Background Buster (50-486-805, Innovex Biosciences, Richmond, CA, USA). Sections were incubated overnight at 4 °C with primary antibodies targeting key neuroinflammatory processes (see Table 1 for antibody details).

To assess inflammation, antibodies against interleukin-6 (IL-6), a pro-inflammatory cytokine [26], and tumor necrosis factor alpha (TNF), a central mediator of inflammatory signaling [27], were used. For innervation, sections were incubated with protein gene product 9.5 (PGP9.5) a pan-neuronal marker [28], and phosphorylated and non-phosphorylated (1:1) neurofilament heavy chain (NFH), to visualize axonal structure and integrity [29]. Immune cell infiltration was evaluated using lymphocyte antigen 6 complex locus C (Ly6C), a marker of monocytes and inflammatory myeloid cells [30], and cluster of differentiation 68 (CD68), a macrophage lineage marker indicative of phagocytic activity [31]. In addition, discs were stained for the neuropeptide Substance P (SubP), given its known role in nociceptive signaling and its relevance as a marker of pain-associated neural activation in degenerated disc tissue [20,21]. Following primary antibody incubation, sections were washed and treated with fluorescently conjugated secondary antibodies (Table 1) and then mounted with DAPI-containing mounting medium (Vector Laboratories, H-2000-10, Newark, CA, USA). Species-specific IgG control (Table 1) and no-primary control-stained tissues were imaged and used as a reference for identifying non-specific primary and secondary antibody binding across tissues (Figures S1 and S2). Goat DRGs, liver, and lymph node tissue were utilized as positive controls (Figures S2 and S3). Imaging was performed using a Zeiss Axio Scan.Z1 slide scanner (Carl Zeiss Microscopy, Oberkochen, Germany). Immunofluorescent images were thresholded, and target deposition/localization was quantified as percent fluorescent area within hand-drawn ROIs within the NP and AF of the disc.

### 2.5. Immunohistochemical Analysis of Spinal Cord and DRG

Spinal cord and DRG tissues were isolated from the cervical motion segments at 12 weeks following ChABC induced degeneration. Samples were fixed, paraffin embedded, and sectioned to 10 µm thickness. Sections were deparaffinized and rehydrated through graded ethanol washes. Antigen retrieval was performed by incubating slides overnight at 60 °C in citrate buffer (Target Retrieval Solution, S169984-2, Agilent, Santa Clara, CA, USA) at pH 6.0. To reduce nonspecific binding, all sections were blocked with Background Buster (50-486-805, Innovex Biosciences, Richmond, CA, USA). Three antibodies were tested in both spinal cord and DRG sections. Sections were incubated overnight at 4 °C with primary antibodies (see Table 1 for antibody details). To identify reactive astrocytes in the spinal cord and satellite glial cells in the DRG, an antibody against Glial fibrillary acidic protein (GFAP) was used. To detect resident microglia in the spinal cord and macrophages or microglial-like cells in the DRG, sections were incubated with anti-ionized calcium-binding adapter molecule 1 (Iba1). Lastly, to assess nociceptive neuropeptide signaling, an antibody against Substance P (SubP) was used. These markers were selected because astrocyte and microglial activation in the spinal cord contribute to central sensitization, while glial activity, immune infiltration, and neuropeptide production in the DRG represent the initiation and amplification of nociceptive signaling [18,19,20,21].

Following primary antibody incubation, sections were washed and treated with fluorescently conjugated secondary antibodies (Table 1) and then mounted with DAPI-containing mounting medium (Vector Laboratories, H-2000-10, Newark, CA, USA). Imaging was performed at high resolution using a Zeiss Axio Scan.Z1 slide scanner under identical exposure settings across groups. Immunofluorescent signals were digitally thresholded, and regions of interest were drawn in the dorsal horn of the spinal cord (300 µm × 300 µm) and within the DRG (1500 µm × 1500 µm). The dorsal horn was selected because it receives majority of sensory input from the periphery, including nociceptive afferent fibers, and is therefore the primary site of sensory processing within the spinal cord. The DRG was analyzed because it serves as the first relay point for sensory afferents, where glial activation, immune infiltration, and neuropeptide release are key events in the initiation of nociceptive signaling [32,33]. Marker presence was quantified as the percent fluorescent area within each region of interest to provide a semi-quantitative measure of localization and relative abundance.

### 2.6. Statistical Analysis

A power analysis was performed to identify significant differences in intervertebral disc structural and functional changes with degeneration (effect size > 0.6, power 0.8, yielding a sample size between n = 3 to n = 9 dependent on outcome) [5]. All statistical analyses were performed using GraphPad Prism 10 (GraphPad Software, Boston, MA, USA). Data distributions were first assessed for normality using the Shapiro–Wilk test. Outcomes from the intervertebral disc (IVD) included biomechanical parameters (toe modulus, compressive modulus, creep strain, compressive strain), MRI-derived NP T2 relaxation times, histological scores, and immunofluorescence outcomes. Disc mechanical parameters and histology scores, which followed normal distributions, were analyzed with one-way ANOVA followed by Tukey’s post hoc test. Immunofluorescence outcomes, quantified as percent fluorescent area for each marker, were analyzed with the Kruskal–Wallis test followed by Dunn’s multiple comparison test. Statistical significance was defined as *p* < 0.05, and trends were defined as *p* < 0.1. All disc-level analyses were stratified by experimental group—control (pooled C2–C3, C3–C4 and C4–C5 discs from animals never receiving spine surgery), adjacent (C3–C4 level from ChABC injected animals), and ChABC (2U and 5U ChABC injected discs). 2U and 5U ChABC discs were pooled for this study, as our prior work demonstrated no detectable differences in disc height reduction or NP T2 relaxation times between these groups [2].

To assess structure–function–neuroinflammatory relationships within the disc, a Pearson correlation matrix related IVD immunofluorescence marker deposition to disc mechanics, NP T2 values, and histological scores, with correlations reported as Pearson’s r. For target marker deposition correlations, the percent fluorescent area was summed across NP and AF regions within IVD samples. Pearson r correlation values (r_p_) between measured variables were interpreted as either low (r_p_ < 0.29), medium (0.3 < r_p_ < 0.49), or high (r_p_ > 0.5) effect size correlations between measured variables. Significance was defined as correlations with *p* < 0.05. For neural tissues, outcomes from the spinal cord and DRG were compared between healthy and ChABC groups using Welch’s t-tests. Because the neural pathways connecting specific discs to the spinal cord and DRG in the goat cervical spine are not defined, tissues from ChABC-injected levels (C2–C3 and C4–C5) and the adjacent level (C3–C4) were grouped together for analysis and compared to spinal levels from control animals not receiving spine surgery.

## 3. Results

### 3.1. ChABC-Induced IVD Degeneration Induces Structural and Mechanical Changes Within Injected and Adjacent IVDs

**Figure 2:**
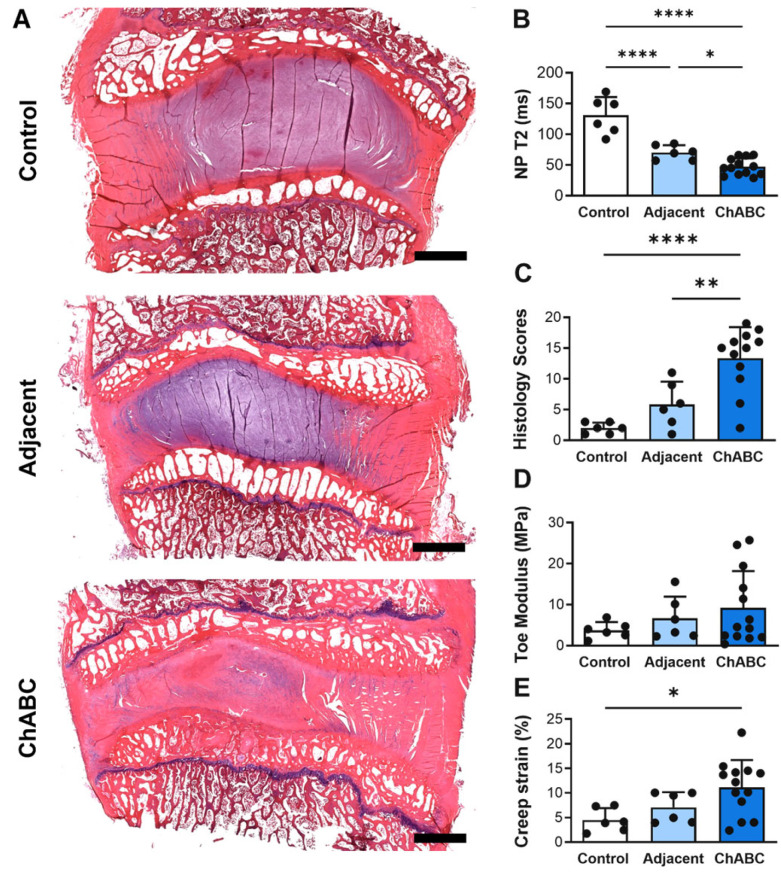
Structural, compositional, and mechanical assessment of intervertebral discs following Chondroitinase ABC (ChABC) injection. (**A**) Representative hematoxylin- and eosin (H&E)-stained mid-sagittal sections of intervertebral discs from a non-surgical control, an adjacent disc, and a ChABC-injected disc, demonstrating progression of intervertebral disc degeneration. Scale bars = 2 mm. (**B**) Quantification of NP T2 relaxation times showing reduced hydration in ChABC-injected discs relative to control and adjacent levels. (**C**) Histological grading of disc degeneration indicating increased degeneration scores in ChABC-injected discs. (**D**) Biomechanical testing revealed increased toe modulus in ChABC-injected discs compared to control and adjacent discs. (**E**) Creep strain was elevated in ChABC injected discs. Data presented as mean ± SD. * *p* < 0.05; ** *p* < 0.01; **** *p* < 0.0001.

Intradiscal injection of ChABC induced a range of structural and mechanical changes within both injected and adjacent to injection IVDs (Figure 2A), reducing NP T2 times within injected IVDs compared to both adjacent to injection IVDs (*p* = 0.0456) and to control IVDs (*p* < 0.0001) harvested from a healthy (non-operated) goat (Figure 2B). Adjacent to injection IVDs also had decreased NP T2 times (*p* < 0.0001) compared to control IVDs (Figure 2B). ChABC injection also produced severe structural degeneration, with increased histological scoring compared to adjacent (*p* = 0.0041) and control IVDs (*p* < 0.0001, Figure 2C). Lastly, ChABC injection significantly increased creep strain compared to control IVDs (*p* = 0.0171, Figure 2D), though no changes in toe moduli were observed. Further, no changes in mechanical properties were observed within the adjacent IVDs compared to controls. Adding to our previous findings of ChABC injection producing structural and mechanical IVD degeneration [5], the incorporation of a control group of IVDs from an unoperated goat extends our evaluations throughout the spine and reveals that adjacent to ChABC-injected IVDs also experience some degree of degenerative change, most notably within NP tissue.

### 3.2. ChABC-Induced IVD Degeneration Promotes Some Nerve Ingrowth and Monocyte Recruitment

**Figure 3:**
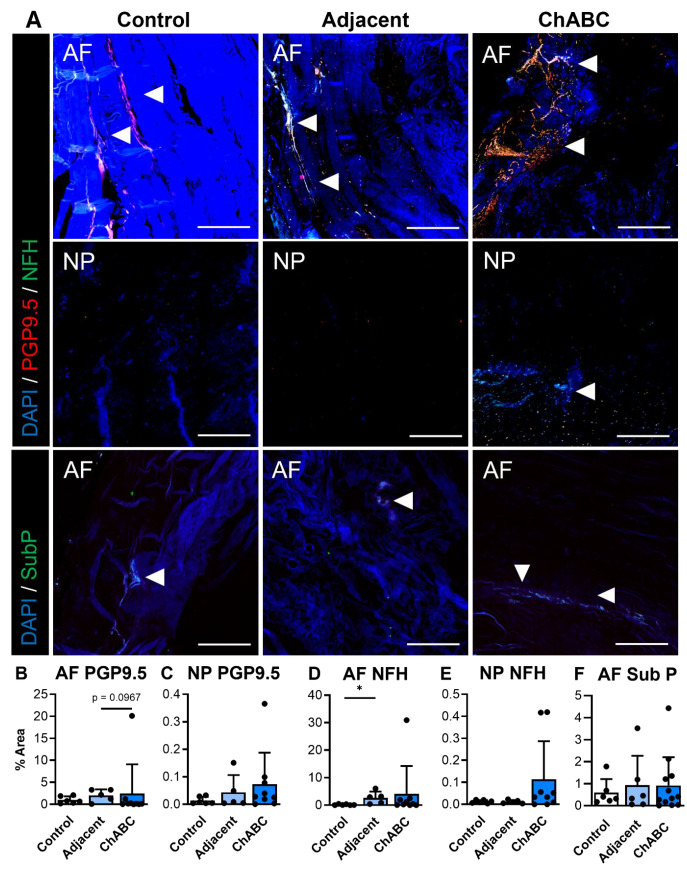
Immunofluorescent visualization and quantification of disc innervation and pain markers. (**A**) Representative immunofluorescence images showing innervation markers PGP9.5 (red) and neurofilament-heavy chain (NFH; green) within the annulus fibrosus (AF) and nucleus pulposus (NP), and the pain-associated neuropeptide Substance P (SubP; green) within the AF. Arrowheads indicate positive staining. Nuclei are counterstained with DAPI (blue). Scale bars = 200 µm. Quantification of the percent area of positive staining for each marker across non-surgical control, adjacent, and ChABC-injected groups: (**B**) PGP9.5 in AF, (**C**) PGP9.5 in NP, (**D**) NFH in AF, (**E**) NFH in NP, and (**F**) Substance P in AF. Data represent mean ± SD, with individual data points shown. * *p* < 0.05.

Extending our analysis beyond structure and function, and to further characterize disease within this model, we next evaluated evidence of innervation and inflammation across our control, adjacent, and ChABC-injected IVDs. With respect to innervation, evidence of nerve markers, PGP9.5 and NFH, was more frequently observed within the AF regions of both adjacent and ChABC-injected IVDs (Figure 3A). Minimal PGP9.5 or NFH deposition was observed throughout AF and NP regions of control IVDs and NP regions from adjacent and ChABC-injected IVDs (Figure 3A). To further assess nociceptive mechanisms in this model, we also stained for the neuropeptide, substance P. Results revealed no evidence of substance P deposition throughout the NP tissues of all groups, while positive substance P staining was only observed in the AF tissue of ChABC-injected IVDs. Quantification of these innervation markers showed that only NFH was increased within adjacent IVDs compared to control (*p* = 0.0389, Figure 3D). No additional statistically significant changes were observed in nerve or neuropeptide marker deposition across groups (Figure 3B,E). Though minimal statistically significant changes were observed, our analysis revealed clear evidence of nerve fibers and neuropeptide activity throughout the outer AF of goat IVDs across groups.

**Figure 4:**
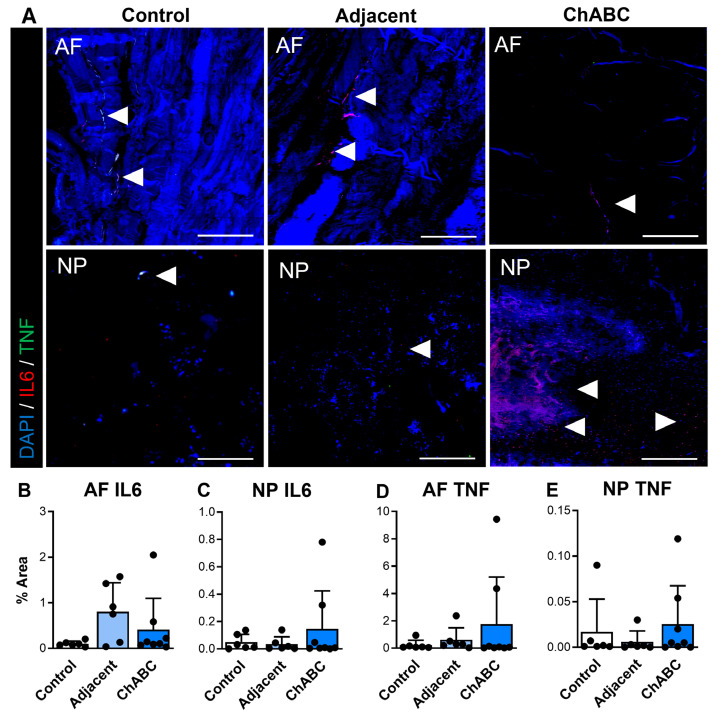
Inflammatory cytokine production within intervertebral discs of non-surgical control, adjacent, and ChABC-injected groups. (**A**) Representative immunofluorescence images showing interleukin-6 (IL-6—red) and tumor necrosis factor-α (TNF—green) staining in the annulus fibrosus (AF) and nucleus pulposus (NP) of non-surgical control, adjacent, and ChABC-injected discs. Nuclei are counterstained with DAPI (blue). White arrowheads indicate regions of positive cytokine deposition. Scale bars = 200 µm. Quantification of the percent area of positive staining for (**B**) IL-6 in the AF, (**C**) IL-6 in the NP, (**D**) TNF in the AF, and (**E**) TNF in the NP. Data are presented as mean ± SD, with each dot representing one sample.

**Figure 5:**
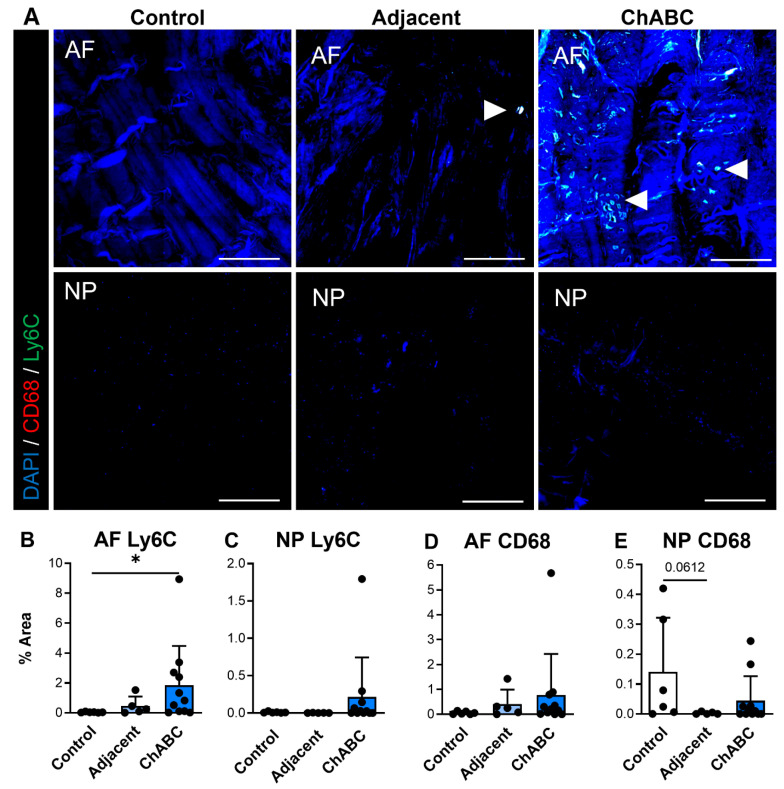
Macrophage-associated marker production within intervertebral discs of non-surgical control, adjacent, and ChABC-injected groups. (**A**) Representative immunofluorescence images showing macrophage marker CD68 (red) and monocyte marker Ly6C (green) staining in the annulus fibrosus (AF) and nucleus pulposus (NP) of non-surgical control, adjacent, and ChABC-injected discs. Nuclei are counterstained with DAPI (blue). White arrowheads indicate positive staining of macrophages or monocyte-like cells. Scale bars = 200 µm. Quantification of the percent area of positive staining for (**B**) CD68 in the AF, (**C**) CD68 in the NP, (**D**) Ly6C in the AF, (**E**) and Ly6C in the NP. Increased CD68 and Ly6C immunoreactivity were observed within the AF of ChABC-injected discs compared to control and adjacent levels, with minimal changes detected in the NP. Data are presented as mean ± SD, with each dot representing one sample. * *p* < 0.05.

We additionally carried out similar staining for inflammatory cytokines and immune cell markers (specifically monocytes and macrophages) across control, adjacent, and ChABC-injected IVDs. Staining for the inflammatory cytokines, IL6 and TNF, was observed more notably within adjacent and ChABC-injected IVDs compared to control, though no significant changes in marker deposition were observed across groups for either marker (Figure 4). Evaluating the common monocyte marker, Ly6C, the AF region of ChABC-injected IVDs had significantly increased Ly6C deposition (*p* = 0.0381) compared to control IVDs (Figure 5A,B). No significant changes in presence of the mature macrophage marker, CD68, were observed across groups (Figure 5). Evaluation of inflammatory markers suggested increased monocyte infiltration occurs within the AF region 12 weeks following ChABC injection. Results also reveal adjacent and control IVDs to have low levels of common inflammatory cytokine production and monocyte/macrophage infiltration.

### 3.3. IVD Innervation and Inflammation Correlate with Structural and Mechanical Changes

**Figure 6:**
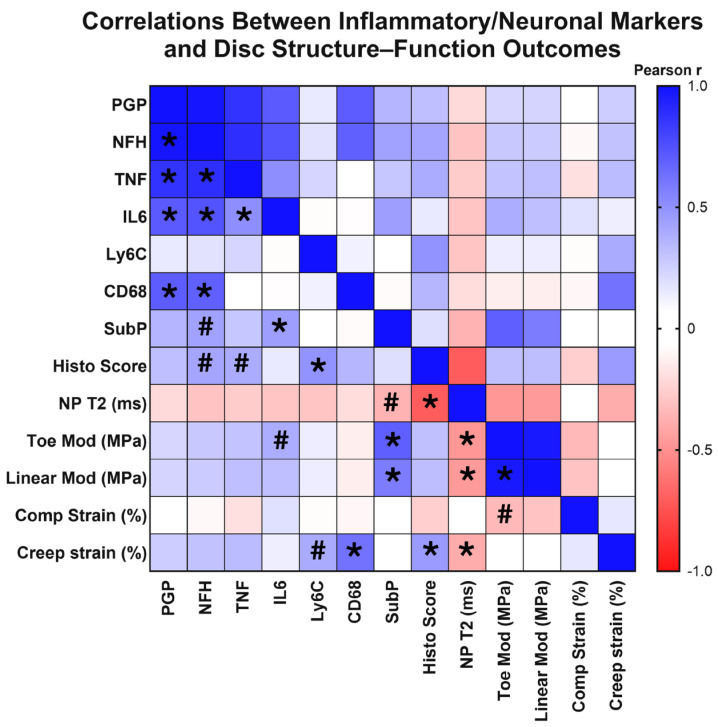
Correlation matrix of structural, inflammatory, and innervation metrics across intervertebral disc samples. Heatmap showing Pearson correlation (r_p_) coefficients among disc mechanical properties, MRI-based composition measures, histological grading, and markers of inflammation, immune cell infiltration, and innervation in the disc. Positive correlations are shown in blue and negative correlations in red, with color intensity proportional to the correlation strength. Asterisks (*) indicate statistically significant correlations (*p* < 0.05) and hash marks (#) denote trends (0.05 < *p* < 0.10). The matrix illustrates relationships among disc mechanical properties, histological grading, inflammatory cytokine levels, and neural marker deposition across all experimental groups.

Across our control, ChABC-injected and adjacent to injection IVDs, we observed a wide range of degeneration, as characterized by our extensive analyses of IVD structure and function. Given this variation in extent of degeneration, we next tested whether structural and functional changes correlated with the extent of inflammation and innervation, as characterized by inflammatory cytokine, monocyte, and macrophage marker, nerve marker, and neuropeptide deposition. First, with respect to innervation markers, results revealed that deposition of the nerve marker, PGP9.5, significantly positively correlated with NFH (*p* < 0.0001, r_p_ = 0.983), IL6 (*p* = 0.002, r_p_ = 0.710), TNF (*p* < 0.0001, r_p_ = 0.866), and CD68 deposition (*p* = 0.001, r_p_ = 0.700) (Figure 6). NFH production positively correlated with IL6 (*p* = 0.001, r_p_ = 0.735), TNF (*p* < 0.0001, r_p_ = 0.885), and CD68 deposition (*p* = 0.002, r_p_ = 0.690) (Figure 6). Interestingly, deposition of the neuropeptide, substance P, significantly positively correlated with functional mechanical property changes, including toe (*p* < 0.0001, r_p_ = 0.693) and linear modulus (*p* = 0.003, r_p_ = 0.577) (Figure 6). Additional associations were observed with a trending positive correlation between NFH deposition and increased histological scores (*p* = 0.068, r_p_ = 0.416) (indicative of more severe IVD degeneration), and a trending negative correlation between substance P deposition with lower, more degenerated, NP T2 values (*p* = 0.079, r_p_ = −0.365) (Figure 6). These correlation analyses revealed highly significant positive correlations between innervation and inflammatory markers, indicating a possible synergistic mechanism. Further, the neuropeptide, substance P, was significantly associated with a degenerative IVD phenotype.

Next, we probed correlations between inflammatory markers, including cytokines and monocyte/macrophage markers. The inflammatory cytokines, TNF and IL6, positively correlated with each other (*p* = 0.022, r_p_ = 0.509) (Figure 6), while presence of the monocyte marker, Ly6C, positively correlated with increased histological scores (*p* = 0.021, r_p_ = 0.488), indicative of worsened degeneration (Figure 6). Further, deposition of the macrophage marker, CD68, positively correlated with increased creep strain (*p* = 0.002, r_p_ = 0.619) (Figure 6). A trending positive correlation was also seen between increased Ly6C production and increased creep strain (*p* = 0.066, r_p_ = 0.399) (Figure 6). Trending positive correlations were also seen with increased TNF and IL6 production and disc histological scores (*p* = 0.077, r_p_ = 0.394) and toe modulus (*p* = 0.082, r_p_ = 0.388), respectively (Figure 6). Taken together, this correlative analysis suggests that the extent of monocyte/macrophage presence within IVD tissues is significantly associated with worsened structural and functional outcomes of IVD health.

Evaluating structure–function correlations across our dataset, NP T2 relaxation times were significantly correlated with a range of mechanical property changes, including toe (*p* = 0.017, r_p_ = −0.475) and linear moduli (*p* = 0.019, r_p_ = −0.465) and creep strain (*p* = 0.050, r_p_ = −0.395) (Figure 6). Decreasing NP T2 relaxation times were also significantly correlated with increased (more degenerative) IVD histological scores (*p* < 0.0001, r_p_ = −0.703) (Figure 6). Additional significant correlations were seen within IVD mechanical properties, with significant positive correlations between toe and linear moduli (*p* < 0.0001, r_p_ = 0.968) (Figure 6). These results revealed associations across IVD mechanical properties, in addition to identifying NP T2 relaxation times to be a strong predictor of overall IVD structure and function within the model.

### 3.4. ChABC Injection Induces Neuroinflammatory and Nociceptive Responses in the Spinal Cord

Following our analysis of the intervertebral disc, we next sought to determine whether the increased innervation, inflammation, and immune cell recruitment observed within the disc were accompanied by corresponding changes in the adjacent neural tissues. Specifically, we examined the DRG and spinal cord to evaluate potential neuroinflammatory and nociceptive responses associated with disc degeneration. As the precise segmental innervation patterns in the goat cervical spine could not be confirmed, ChABC-injected and adjacent discs were grouped together and classified as “degenerated,” based on consistent evidence of structural and biochemical degeneration across both levels, and compared to non-operative control spines. This approach allowed for assessment of how disc pathology influences glial activation, immune signaling, and nociceptive marker production within the sensory pathways innervating the affected motion segments. We first investigated the spinal cord, focusing on the dorsal horn, the principal site of sensory processing and nociceptive signal integration.

**Figure 7:**
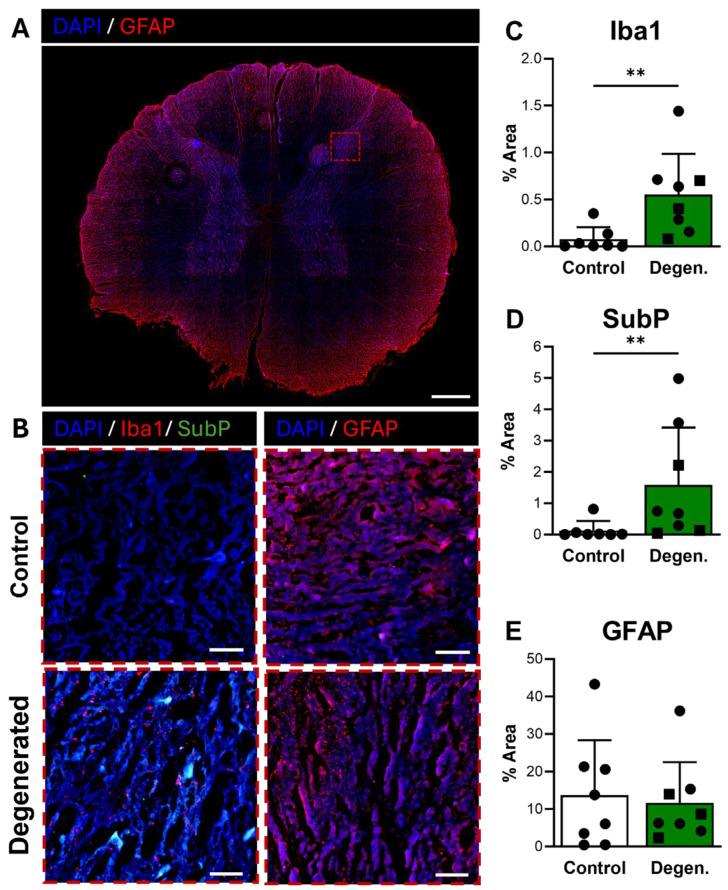
Neuroinflammatory and pain-associated marker deposition in the spinal cord following ChABC injection. (**A**) Representative low-magnification image of a spinal cord cross-section stained for GFAP (red) to visualize astrocyte activation, with the region of interest in the dorsal horn indicated by a dashed box. Scale bar = 500 µm. (**B**) Higher magnification images of the dorsal horn showing Iba1 (red) with Substance P (SubP, green) with DAPI (blue) for microglial activation and pain-associated neuropeptide deposition and GFAP (red) for astrocyte reactivity and in non-surgical control, degenerated groups. Scale bars = 50 µm. Quantification of percent area in the dorsal horn for positive staining of (**C**) Iba1, (**D**) SubP, and (**E**) GFAP. In the degenerated group, circles represent spinal cords segments that were adjacent to ChABC-injected discs, and squares represent spinal cord segments from discs adjacent to the ChABC-injected levels. Data are presented as mean ± SD, with each dot/square representing one sample. ** *p* < 0.01.

Microglial activation, identified by Iba1 immunoreactivity, was broadly distributed in the dorsal horn of the spinal cord (Figure 7A) and appeared elevated in ChABC-injected animals compared to non-operative controls (Figure 7B,C). Quantification confirmed a significant increase in Iba1 expression in degenerative spines compared to controls (*p* = 0.0037) (Figure 7C), consistent with a central neuroinflammatory response. Substance P also showed increased expression in the dorsal horn of ChABC-injected animals (Figure 7B,D). Quantitative analysis demonstrated a significant elevation in SubP expression relative to non-operative controls (*p* = 0.0093) (Figure 7D). These findings indicate that disc degeneration enhances nociceptive signaling in parallel with microglial activation in the spinal cord. GFAP immunoreactivity, which reflects activation of astrocytes and satellite glial cells, remained unchanged between groups (Figure 7B,E).

### 3.5. ChABC Injection Induces Glial Cell Activation in the DRG

**Figure 8:**
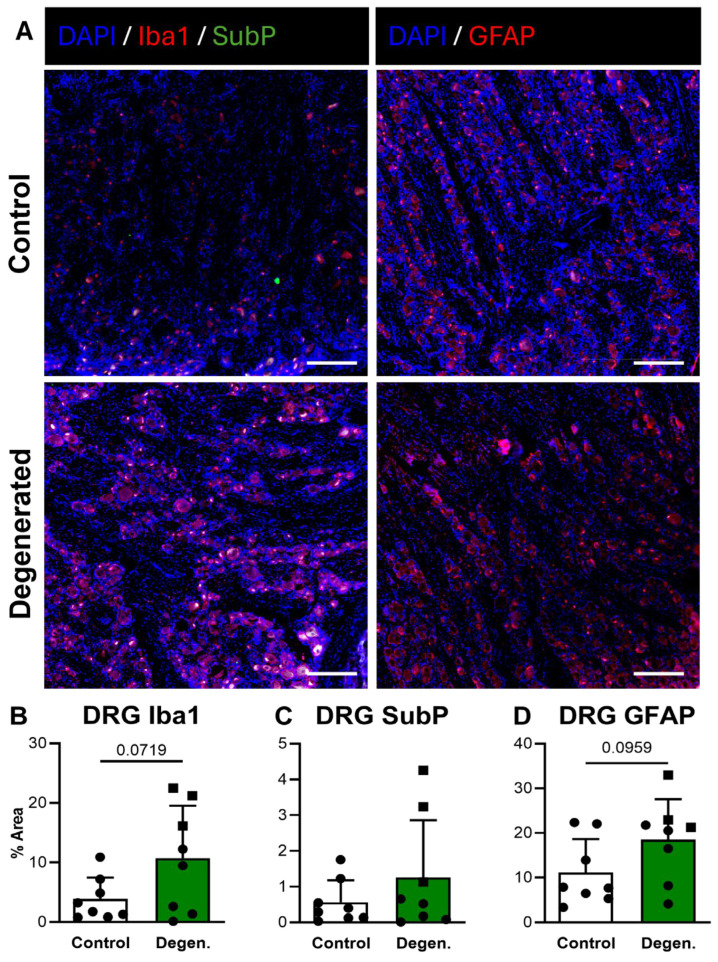
Neuroinflammatory and pain-associated marker deposition in DRG following ChABC injection. (**A**) Representative immunofluorescence images showing Iba1 (red) with Substance P (SubP; green) with DAPI (blue) for macrophage/microglial activation and pain-associated neuropeptide deposition and GFAP (red) for satellite glial cell activation in non-surgical control, and degenerated groups. Scale bars = 50 µm. Quantification of percent area for positive staining of (**B**) Iba1, (**C**) SubP, and (**D**) GFAP in DRGs. In the degenerated group, circles represent spinal cords segments that were adjacent to ChABC-injected discs, and squares represent spinal cord segments from discs adjacent to the ChABC-injected levels. Data are presented as mean ± SD, with each dot/square representing one sample.

In the DRG, Iba1 GFAP immunoreactivity both appeared increased in ChABC-injected animals compared to non-operative controls (Figure 8A,B,D). Quantification revealed trending increases for both markers, although these did not reach statistical significance (Figure 8B,D). There were no statistically significant differences in SubP deposition in the DRG between experimental groups (Figure 8C). Despite the absence of statistical significance, the consistent upward trends in GFAP and Iba1 deposition suggest that disc degeneration promotes localized glial activation within the DRG.

## 4. Discussion

This study sought to determine whether biological drivers of inflammation and pain commonly associated with IVDD in humans are present within a large animal model of ChABC-induced IVDD. Utilizing our previously established goat model of IVDD [5], we analyzed IVDs and neural tissue isolated from non-ChABC-injected control goats and from goats that had received multi-level IVD ChABC injections 12 weeks prior. In addition to a comprehensive analysis of IVD structure and function, our analyses focused on quantifying inflammatory and neuronal protein deposition across spinal tissues. Correlating the extent of inflammatory and neuronal protein deposition with common metrics of IVD health, we identified significant associations between the extent of neuroinflammation and structural and functional features of IVD health. Notably, we saw the extent of IVD monocyte (Ly6C) and macrophage (CD68) marker deposition to significantly correlate with worsened histological scoring and with compromised viscoelastic mechanical properties (creep strain). Similar correlations were observed for innervation, where the extent of the neuropeptide, Substance P, in IVD tissue significantly correlated with worsened compressive mechanical properties, specifically toe and linear moduli. Extending our analysis to nearby neural tissue, we further revealed that ChABC-induced disc degeneration increases neuroinflammatory activation, with increases in both the microglia marker, Iba1, and the neuropeptide, Substance P, within spinal cord tissue. Similarly, we observed trending increases in microglia marker deposition within DRG tissue from goats with degenerative discs. This is noteworthy because satellite glial cells and resident immune cells in the DRG are critical mediators of peripheral sensitization and can amplify nociceptive signaling from the degenerating discs. Collectively, our data demonstrates that enzymatically induced IVD degeneration elicits a cascade of neuroinflammatory responses across spinal tissues.

In our analysis of IVD health, quantified by histological scoring, MRI, and assessment of mechanical properties, we found that ChABC-injection to multiple IVD levels also initiated mild structural and mechanical degeneration within the non-injected adjacent IVDs. These findings support observations in human patients that have identified disease in one level to be a possible risk factor for degeneration at nearby adjacent levels [13] and further emphasize the importance of incorporating appropriate baseline comparator discs when assessing inflammatory and degenerative outcomes in large animal models. Alongside adjacent IVD structural and mechanical changes, we identified increased innervation, as evidenced by NFH deposition, within adjacent IVD AF tissue, suggesting that neuroinflammatory remodeling extends beyond the directly injured discs. Collectively, these findings reinforce the concept that spinal joint disease occurs regionally rather than in isolation and highlights the necessity of distinguishing baseline, non-operated IVDs from adjacent, non-injected discs when evaluating disease progression. This distinction is particularly informative for the future utilization of this large animal model in therapeutic studies, as it provides a more nuanced baseline for identifying potential improvements in both neuroinflammatory and structural features within mildly affected adjacent discs.

Beyond adjacent IVDs and neural tissues, prior studies have reported neuroinflammatory activation across additional spinal tissues, such as lumbar facet joints and spinal ligaments. These clinical investigations suggest that the presence of lumbar synovial cysts [34] and synovial neovascularization (VEGF), innervation (NF-M, NGF, TrkA), and immune cell infiltration (CD11b) are likely contributors to symptomatic pain [35]. Additional clinical insights reveal evidence of neuroinflammation throughout spinal ligaments, where macrophage infiltration (CD68, MHC-II) and increased angiogenesis (CD31) have been observed within diseased, hypertrophied ligamentum flavum [36], in addition to evidence of innervation, identified via neurofilament protein production, within supraspinous and interspinous ligaments [37]. Though beyond the scope and initial design of this study, we have previously reported structural and mechanical degenerative changes to facet joint cartilage within IVD ChABC-injected goats [5], and thus, future studies are underway to expand our neuroinflammatory evaluations within the ChABC model to include additional spinal tissues.

In addition to associations between neuroinflammatory markers and IVD structure and mechanics, we identified significant positive associations across biological outcomes. Specifically, we saw increased production of innervation markers PGP9.5 and NFH to be correlated with both inflammatory mediators (IL6, TNF) and with monocyte and macrophage markers. Increased inflammatory cytokines, macrophage infiltration, and innervation are all features of human IVDD [6,38,39]. Our results provide new insights into similar inflammatory and neuronal biological signatures that are features of IVDD within the goat cervical spine. These results also mimic the extensive prior investigations in preclinical small animal models of IVDD, where increased inflammatory mediator expression, macrophage infiltration, and neuronal activation are all identified downstream of induced IVD injury and disease [10,11,14,15,40]. Specifically, we identified significant increases in the monocytic marker, Ly6C, within the AF of ChABC-injected IVDs, as well as significant correlations between monocyte and macrophage markers, with IVD structural and functional decline. These increases in monocytic markers were most apparent within AF regions, even though the chemical insult was localized to the NP, suggesting that the loss of GAGs in the NP elicits a change in the nearby AF tissues that may facilitate innervation and immune cell ingrowth to occur. Furthermore, our correlative findings between macrophage marker deposition and structural and mechanical decline, as well as neuronal marker deposition, strongly coincide with the known function of these innate immune cells in responding to tissue damage by propagating further inflammation and innervation and driving tissue catabolism [41]. Ultimately, these results provide substantial rationale for future immune-targeted exploration, which can aim to define a causal rather than correlative relationship between the presence of monocytes and macrophages and IVD degeneration and pain.

In addition to disc-level changes, we observed neuroimmune activation in adjacent neural tissues, which supports the notion that degeneration of the intervertebral disc can drive downstream responses in the DRG and spinal cord. The dorsal horn of the spinal cord is broadly recognized as the primary site receiving the majority of peripheral sensory input, including nociceptive afferent fibers, and as the central hub for integration and processing of somatosensory signals [42,43]. Similarly, the DRG serves as the first relay station for sensory afferent neurons in the periphery, where glial and immune activation, macrophage/monocyte infiltration, and neuropeptide release are critical early steps in the initiation of nociceptive signaling [44,45,46]. The elevated Iba1 and Substance P in the dorsal horn, and trending GFAP and Iba1 shifts in the DRG, suggest that disc degeneration may activate both central and peripheral neuroimmune pathways. These responses reinforce the concept that disc degeneration is not simply a local tissue event but may provoke sensitization and immune–neural crosstalk in sensory pathways.

Comparable findings have been reported in small animal models. In a rat annulus fibrosus injury model, Lai et al. [12] demonstrated that lumbar disc AF puncture produced acute increases in Substance P, Iba1, and GFAP deposition within the DRG and dorsal horn, followed by a chronic astroglial response that persisted up to eight weeks post injury. Other rodent studies similarly describe dorsal-horn microglial and astrocytic activation (Iba1 and GFAP) and DRG sensitization with increased nociceptive neuropeptides (Substance P, CGRP) after disc or nerve injury [47,48,49,50,51]. Consistent with these reports, our study identified microglial activation and heightened Substance P deposition within the dorsal horn of degenerated spines, as well as glial activation trends in the DRG. However, whereas Lai et al. observed a transient early microglial response that resolved over time, our data indicate a persistent activation state at twelve weeks, suggesting a sustained neuroinflammatory environment in a larger, more chronic model. Bearing similarities with prior studies, it is important to note that the AF puncture approach produces a physical avenue for innervation and immune cell trafficking as well as the the release of NP material into the spinal cord and DRG space, which itself will produce an inflammatory response. The ChABC approach utilized within this study does not rupture the AF and thus may better recapitulate what happens clinically during IVDD in the absence of AF rupture. Collectively, while small animal models have established the mechanistic link between disc injury and central/peripheral neuroinflammation, our study is the first to demonstrate these coordinated DRG and spinal-cord neuroimmune responses in a large animal model of intervertebral disc degeneration, thereby bridging translational understanding between rodent and large animal models.

Because precise segmental innervation patterns in the goat cervical spine are not yet established, we grouped the ChABC-injected discs and their adjacent discs into a combined “degenerated” category for our analysis of neural tissues. We recognized that although both disc categories showed clear structural and biochemical evidence of degeneration, we cannot definitively identify which specific DRGs innervate which discs. Indeed, retrograde tracing studies in small animal models have shown that intervertebral discs may receive sensory inputs from multiple DRG levels (e.g., Th13–L6 in rats) rather than a single level mapping [52]. Furthermore, prior work in the same large animal model has shown that ChABC injection in discs also induces degeneration of adjacent facet joints [5], raising the possibility that neuroinflammatory responses in DRGs or spinal cord may in part reflect afferent activation from the facets in addition to the discs. Future retrograde nerve-tracing experiments will be required to exactly map DRG-disc and DRG-facet innervation in the cervical goat spine, enabling clearer attribution of neural responses to specific pathologies in the motion segment.

Limitations to the presented study should be acknowledged. First, while this study demonstrates associations between disc degeneration and neuroinflammatory activation following ChABC injection, a sham needle puncture control group was not included. This choice was supported by our prior studies using this same ChABC goat model, which incorporated sham saline injection groups specifically to isolate the effects of needle insertion and fluid delivery [2,53]. In those studies, sham injection produced no significant differences relative to non-injected control IVDs across structural, functional, mechanical, or histopathological outcomes, nor did it alter inflammatory cytokine or catabolic enzyme expression. Nonetheless, because these prior studies did not specifically assess neuroinflammatory markers and did not include the more rigorous baseline group of non-operated goats, we cannot definitively exclude the possibility that needle puncture alone induces a mild neuroinflammatory response. Second, the current work represents a secondary analysis of samples generated for primary studies focused on characterizing functional and mechanical disc-level changes following ChABC injection [5]. As a result, some neuroinflammatory and immune-related outcome measures may be underpowered, potentially limiting the detection of more subtle effects. Within large-animal studies, sample size remains an inherent limitation due to logistical, ethical, and financial constraints. Despite these limitations, the consistency of the observed relationships between disc pathology, immune cell markers, neural innervation, and spinal cord activation supports the biological relevance of the findings and underscores the translational value of this large-animal IVDD model.

In summary, this study demonstrates that ChABC-induced degeneration in the goat cervical spine reproduces key biological and neuroinflammatory features of human IVDD, including monocyte/macrophage infiltration, cytokine upregulation, and increased innervation throughout ChABC-injected and adjacent IVDs. The neuroinflammation observed in the DRG and spinal cords of degenerative spines also provides a promising surrogate measure of pain in large animal models. The observed associations between these molecular changes and declines in disc structure and function provide insight into possible inflammation-driven mechanisms that underlie spine disease and its associated pain. Importantly, the evidence of adjacent-level disc degeneration in this model and inflammatory activation within corresponding neural tissues further aligns with clinical observations of spinal degeneration as a complex multi-factorial disease and supports the utility of this model for testing emerging immune- and neuroinflammation-targeted therapies aimed at slowing degeneration and alleviating pain.

## Figures and Tables

**Table 1:** Antibodies utilized to assess inflammation, immune cell infiltration, and neural markers in the intervertebral disc, DRG, and spinal cord (SC).

Antibody	Vendor (Cat#)	Antigen Retrieval	Dilution	Secondary Antibody	Dilution	Tissue
PGP9.5	Millipore (Darmstadt, Germany) (SAB4503057)	Proteinase K	1:100	Abcam (Cambridge, UK) (ab150064)	1:500	IVD
NFH	BioLegend (San Diego, CA, USA), 1:1(801601, 801701)	Proteinase K	1:1000	Invitrogen (Carlsbad, CA, USA) (A11029)	1:250	IVD
IL6	Abcam (ab6672)	Proteinase K	1:250	Abcam (ab150064)	1: 500	IVD
TNF	Biorbyt (Cambridge, UK) (orb432637)	Proteinase K	1:250	Abcam (ab150153)	1:250	IVD
Ly6C	Abcam (ab25377)	Proteinase K	1:200	Abcam (ab150153)	1:250	IVD
CD68	Abcam (ab213363)	Proteinase K	1:200	Abcam (ab150064)	1:500	IVD
GFAP	Abcam (ab7260)	Citrate buffer	1:500	Abcam (ab150064)	1:500	SC & DRG
Iba1	Abcam (ab178846)	Citrate buffer	1:500	Abcam (ab150064)	1:500	SC & DRG
Substance P	Abcam (ab14184)	Citrate buffer	1:500	Invitrogen (A11029)	1:250	SC & DRG
Rabbit IgG	Abcam (ab172730)	Proteinase K/ Citrate buffer	1:650	Abcam (ab150064)	1:500	IVD, SC & DRG
Rat IgG	Abcam (ab37361)	Proteinase K	1:250	Abcam (ab150153)	1:250	IVD
Mouse IgG	BioLegend (401401)	Proteinase K/ Citrate buffer	1:500	Invitrogen (A11029)	1:250	IVD, SC & DRG

## Data Availability

All data is included in the manuscript or Supplemental Files.

## Supplementary Materials

The following supporting information can be downloaded at https://www.mdpi.com/article/10.3390/cells15030286/s1.

## Abbreviations

The following abbreviations are used in this manuscript:

IVD	Intervertebral disc
IVDD	Intervertebral disc degeneration
NP	Nucleus pulposus
AF	Annulus fibrosus
DRG	Dorsal root ganglion
SC	Spinal cord
MRI	Magnetic resonance imaging
ROI	Region of interest
ChABC	Chondroitinase-ABC
IL-6	Interleukin-6
TNFα	Tumor necrosis factor alpha
NFH	Neurofilament (heavy and light chain)
Ly6c	Lymphocyte antigen 6 complex locus C
CD68	Cluster of differentiation 68
SubP	Substance P
GFAP	Glial fibrillary acidic protein
Iba1	Ionized calcium-binding adaptor molecule 1
