# Supplementary material for: Neuroimmune Activation in a Goat Model of Intervertebral Disc Degeneration
Source: Cells. 2026 Feb 3;15(3):286. doi: 10.3390/cells15030286 (PMC12896899; doi:10.3390/cells15030286)
Supplement: Supplementary file 1 [file cells-15-00286-s001.zip › cells-4103210-supplementary.pdf]

Supplemental Figures

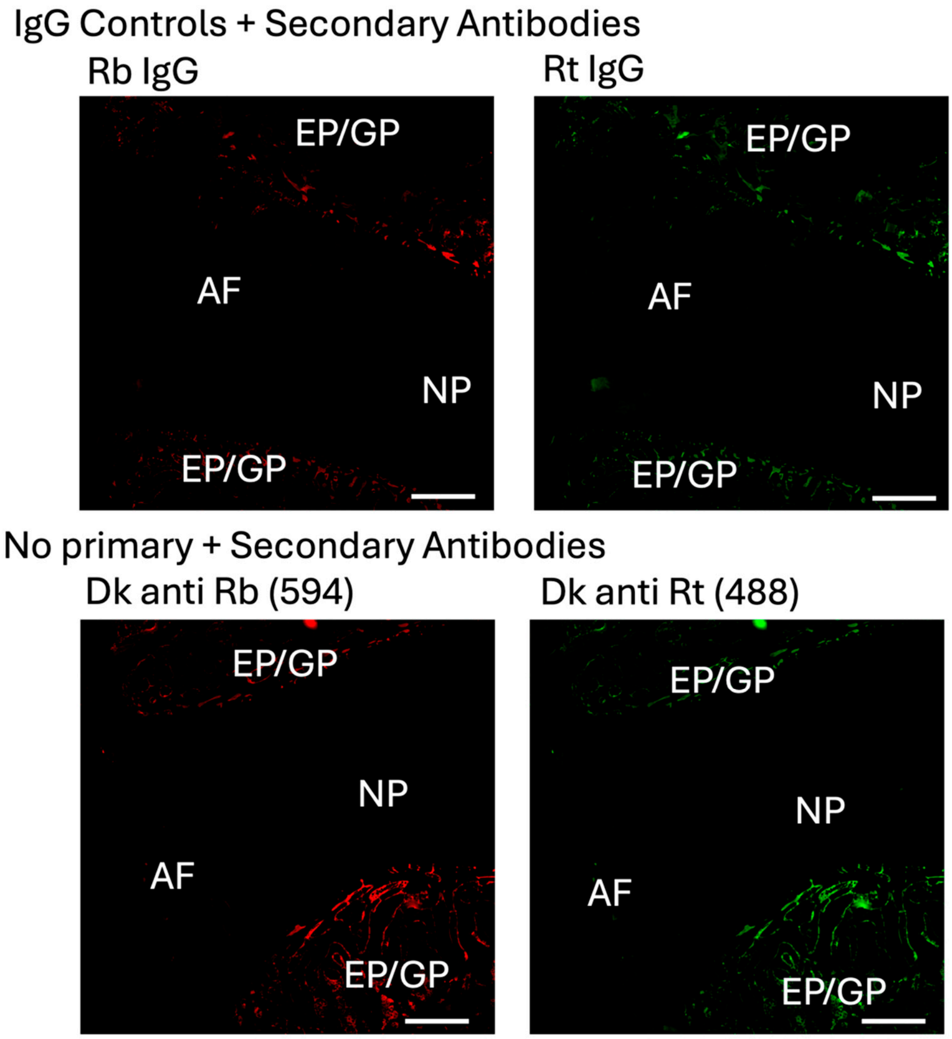

Supplemental Figure S1: Control staining for intervertebral disc tissue. Representative fluorescence images of intervertebral disc sections processed with isotype IgG controls or without primary antibodies, demonstrating minimal nonspecific signal. Images show the annulus fibrosus (AF), nucleus pulposus (NP), and cartilaginous endplate/growth plate region (EP/GP). Red and green channels correspond to secondary antibodies used for experimental markers, with no specific immunoreactivity detected in control conditions. Scale bars = 1,000 μm.

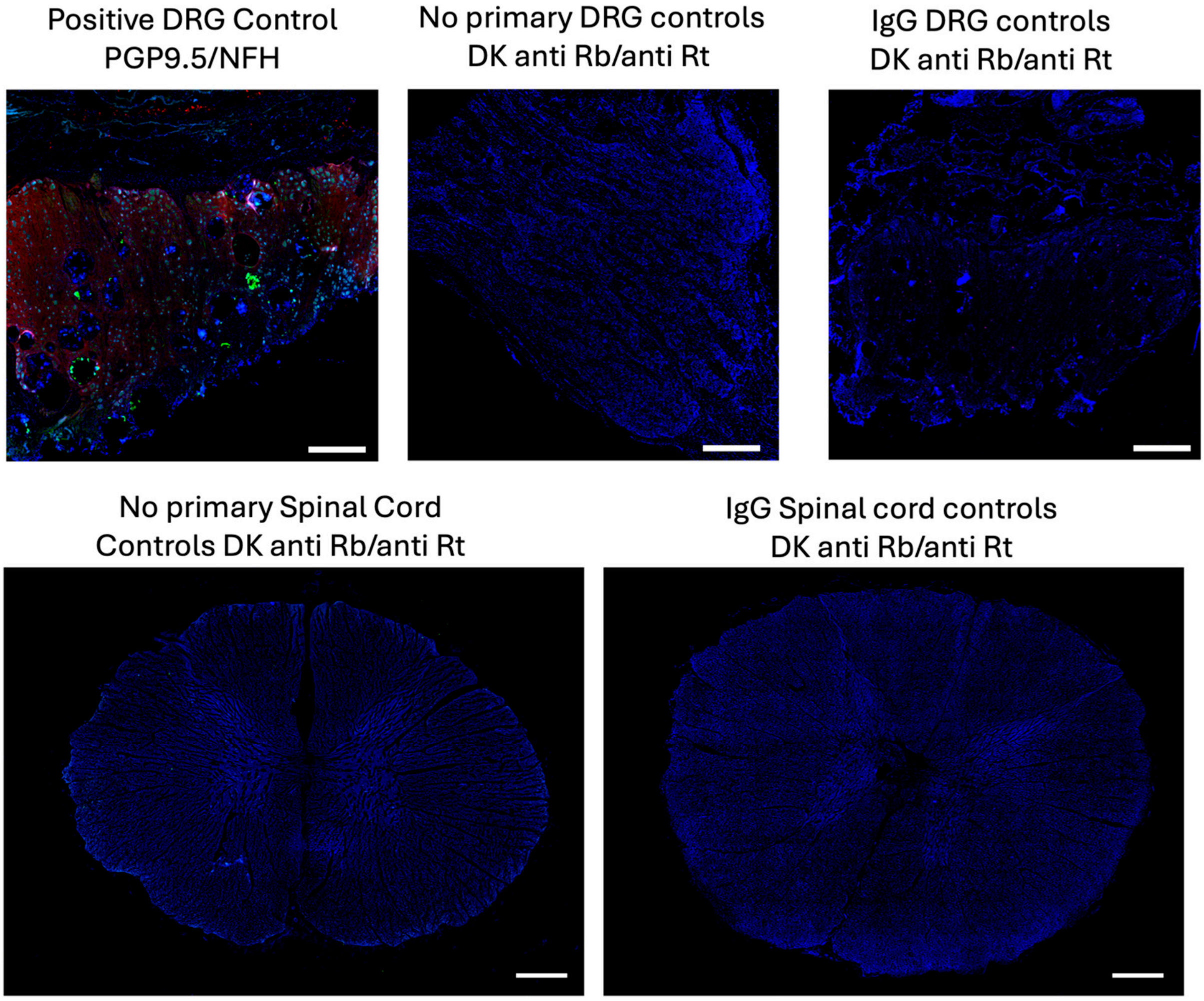

Supplemental Figure S2: Control staining for dorsal root ganglia and spinal cord tissues. Representative images demonstrating the absence of specific signal in dorsal root ganglia (DRG) and spinal cord sections processed without primary antibodies or with isotype IgG controls. Sections were counterstained with DAPI (blue) to visualize nuclei. No specific immunoreactivity is observed, confirming minimal nonspecific binding and low background fluorescence under the imaging conditions used for experimental samples. Scale bars = 500μm.

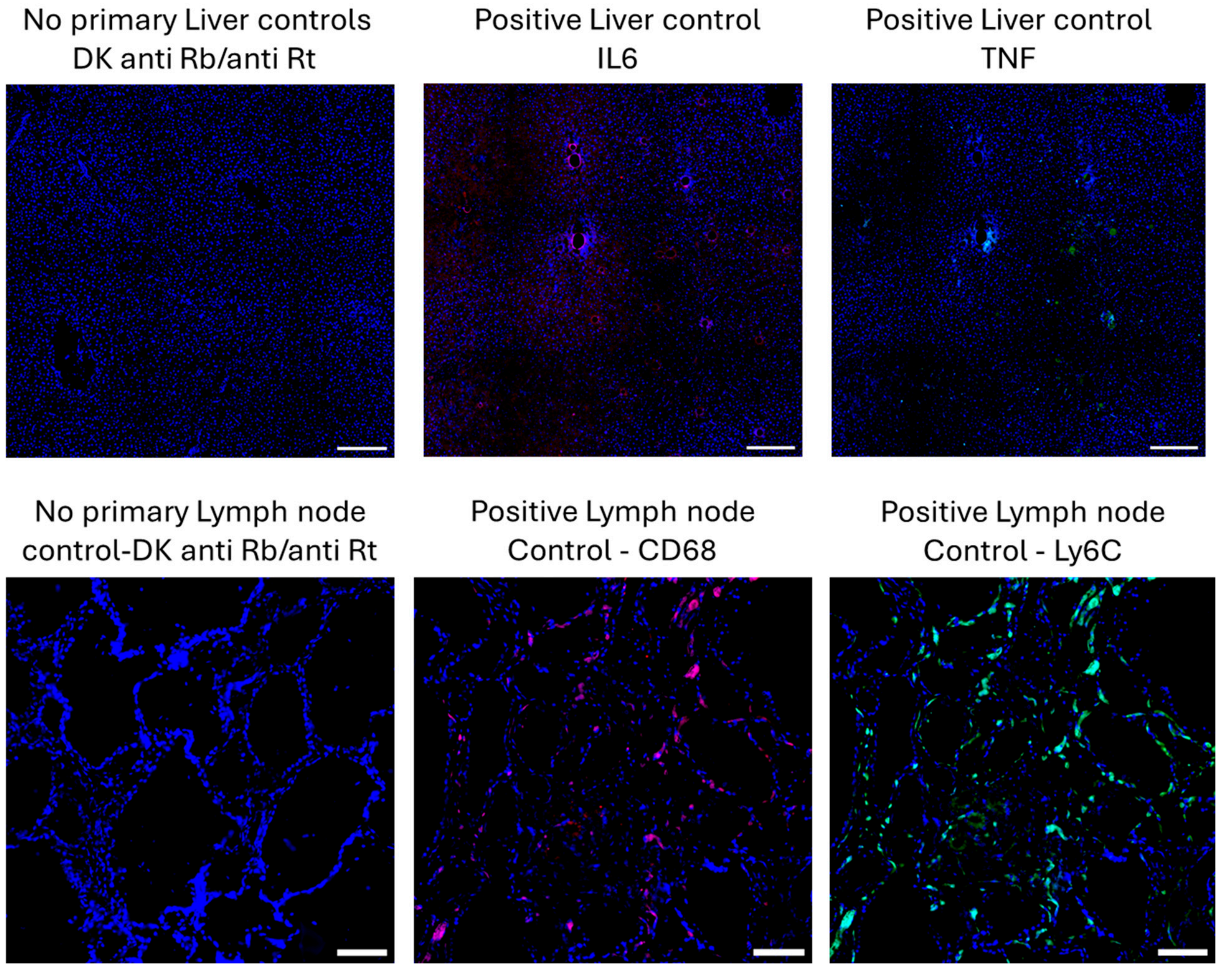

Supplementary Figure S3. Positive control staining of goat liver and lymph node tissues. Representative immunofluorescence images of goat liver and submandibular lymph node sections showing expected staining of inflammatory and immune cell markers IL-6, TNF, CD68, and Ly6C. Sections processed without primary antibodies served as negative controls, and liver and lymph node tissues served as positive controls. All sections were counterstained with DAPI (blue) to visualize nuclei. Scale bars = 200 µm (liver) and 100 µm (lymph node).
